# Clofazimine pharmacokinetics in HIV‐infected adults with diarrhea: Implications of diarrheal disease on absorption of orally administered therapeutics

**DOI:** 10.1002/psp4.13092

**Published:** 2024-01-02

**Authors:** Cindy X. Zhang, Thomas M. Conrad, David Hermann, Melita A. Gordon, Eric Houpt, Pui‐Ying Iroh Tam, Khuzwayo C. Jere, Wilfred Nedi, Darwin J. Operario, Jacob Phulusa, Gerald V. Quinnan, Leigh A. Sawyer, Lynn K. Barrett, Herbert Thole, Neema Toto, Wesley C. Van Voorhis, Samuel L. M. Arnold

**Affiliations:** ^1^ Department of Pharmaceutics University of Washington Seattle Washington USA; ^2^ Emmes Rockville Maryland USA; ^3^ Bill & Melinda Gates Foundation Seattle Washington USA; ^4^ Paediatrics and Child Health Research Group Malawi‐Liverpool Wellcome Trust Clinical Research Programme Blantyre Malawi; ^5^ Institute of Infection and Global Health University of Liverpool Liverpool UK; ^6^ Division of Infectious Diseases and International Health University of Virginia Charlottesville Virginia USA; ^7^ Liverpool School of Tropical Medicine Liverpool UK; ^8^ Center for Emerging and Re‐emerging Infectious Diseases University of Washington Seattle Washington USA; ^9^ Present address: AstraZeneca Rockville MD USA; ^10^ Present address: World Health Organization Suva Central Fiji

## Abstract

Oral drug absorption kinetics are usually established in populations with a properly functioning gastrointestinal tract. However, many diseases and therapeutics can alter gastrointestinal physiology and cause diarrhea. The extent of diarrhea‐associated impact on drug pharmacokinetics has not been quantitatively described. To address this knowledge gap, we used a population pharmacokinetic modeling approach with data collected in a phase IIa study of matched human immunodeficiency virus (HIV)–infected adults with/without cryptosporidiosis and diarrhea to examine diarrhea‐associated impact on oral clofazimine pharmacokinetics. A population pharmacokinetic model was developed with 428 plasma samples from 23 HIV‐infected adults with/without *Cryptosporidium* infection using nonlinear mixed‐effects modeling. Covariates describing cryptosporidiosis‐associated diarrhea severity (e.g., number of diarrhea episodes, diarrhea grade) or HIV infection (e.g., viral load, CD4+ T cell count) were evaluated. A two‐compartment model with lag time and first‐order absorption and elimination best fit the data. Maximum diarrhea grade over the study duration was found to be associated with a more than sixfold reduction in clofazimine bioavailability. Apparent clofazimine clearance, intercompartmental clearance, central volume of distribution, and peripheral volume of distribution were 3.71 L/h, 18.2 L/h (interindividual variability [IIV] 45.0%), 473 L (IIV 3.46%), and 3434 L, respectively. The absorption rate constant was 0.625 h^−1^ (IIV 149%) and absorption lag time was 1.83 h. In conclusion, the maximum diarrhea grade observed for the duration of oral clofazimine administration was associated with a significant reduction in clofazimine bioavailability. Our results highlight the importance of studying disease impacts on oral therapeutic pharmacokinetics to inform dose optimization and maximize the chance of treatment success.

**Study Highlights**

**WHAT IS THE CURRENT KNOWLEDGE ON THE TOPIC?**

Although clofazimine's pharmacokinetic and safety profiles have been studied in healthy volunteers, leprosy patients, and multi‐drug‐resistant tuberculosis patients, no study has been conducted for patients with diarrhea‐related symptoms, and it is unclear whether diarrhea should be considered when dosing clofazimine for cryptosporidiosis.

**WHAT QUESTION DID THIS STUDY ADDRESS?**

This study addressed the question whether an individual with diarrhea dosed orally with clofazimine may experience lower than expected clofazimine exposure.

**WHAT DOES THIS STUDY ADD TO OUR KNOWLEDGE?**

To the best of our knowledge, this is the first study to quantitatively describe a diarrhea‐associated effect on oral drug pharmacokinetics, which has been overlooked.

**HOW MIGHT THIS CHANGE DRUG DISCOVERY, DEVELOPMENT, AND/OR THERAPEUTICS?**

Diarrhea‐associated reduction in oral drug absorption, such as by a magnitude of more than sixfold as demonstrated by this study, may result in patients with diarrhea receiving subtherapeutic doses of drug, leading to treatment failure as well as valuable time and resources being lost. This study highlights the critical need for additional studies to evaluate the impact diarrhea may have on oral drug absorption.

## INTRODUCTION

Oral drug delivery is the most common and preferred route of drug administration because of its ease of administration, noninvasiveness, high compliance, and cost‐effectiveness. Conditions in the gastrointestinal (GI) tract, such as motility, transit time, luminal pH, fluid composition, absorptive surface area, and permeability may have a profound impact on oral drug release, dissolution and permeation, reflected by changes in oral drug bioavailability.^1^, ^2^, ^3^, ^4^, ^5^, ^6^ Although not studied in detail, it is plausible that dosing recommendations based on data collected from populations with typical, healthy GI physiology may result in unexpected changes in drug exposure and subtherapeutic responses in populations presenting with diarrhea.

Observations of potential diarrhea‐associated changes in drug exposure have indeed been reported in literature. A study in Brazil found that antiretroviral stavudine and didanosine plasma levels were substantially lower in human immunodeficiency virus (HIV)–infected patients with diarrhea or wasting compared with HIV‐infected patients without diarrhea.^7^ However, limitations to this study such as variable antiretroviral regimens, doses, and durations, significant body mass index differences between patient groups, and the lack of in‐depth statistical analyses precluded the study from conclusively proving that diarrhea had a significant role in influencing antiretroviral absorption in the study population. Other studies of diarrhea and oral drug pharmacokinetics (PK) yielded mixed results; although some observed potential relationships between diarrhea and reduced plasma drug levels,^8^, ^9^ others did not find any significant diarrhea effect.^10^, ^11^, ^12^

The potential impact of cryptosporidiosis, a diarrheal disease caused by *Cryptosporidium* infection, on drug disposition has not been previously studied.^13^, ^14^, ^15^ Clinical symptoms of cryptosporidiosis range from watery diarrhea, abdominal pain, dehydration, nausea, vomiting, and fever in immunocompetent subjects to persistent diarrhea, severe weight loss, malnutrition, and mortality in malnourished children and immunocompromised persons.^14^, ^16^, ^17^, ^18^ It was estimated that *Cryptosporidium* infection resulted in more than 48,000 deaths and more than 4.2 million disability‐adjusted life‐years lost in 2016 alone.^19^ Clofazimine, an orally administered antimicrobial approved by the US Food and Drug Administration (FDA) to treat leprosy and a “Group B" drug recommended by the World Health Organization to treat multi‐drug‐resistant (MDR) tuberculosis (TB), was shown to be effective in controlling *Cryptosporidium* infection in preclinical in vitro and in vivo models.^20^, ^21^, ^22^ Although clofazimine's PK and safety profiles had been studied in healthy volunteers, leprosy patients, and MDR‐TB patients, no study had been conducted for cryptosporidiosis patients until a phase IIa trial was conducted in Malawi in HIV‐infected adults.^23^, ^24^, ^25^, ^26^, ^27^ The trial employed a unique two‐part design by which clofazimine PK data were collected from HIV‐infected adults with or without *Cryptosporidium* infection matched based on weight, sex, and age.^23^, ^24^ This enabled our study to quantitatively characterize cryptosporidiosis‐associated diarrhea impact on oral clofazimine PK through a population PK modeling approach, underlining the importance of studying potential diarrhea impact on oral drug PK for dose optimization and treatment success.

## METHODS

### Study design

**FIGURE 1:**
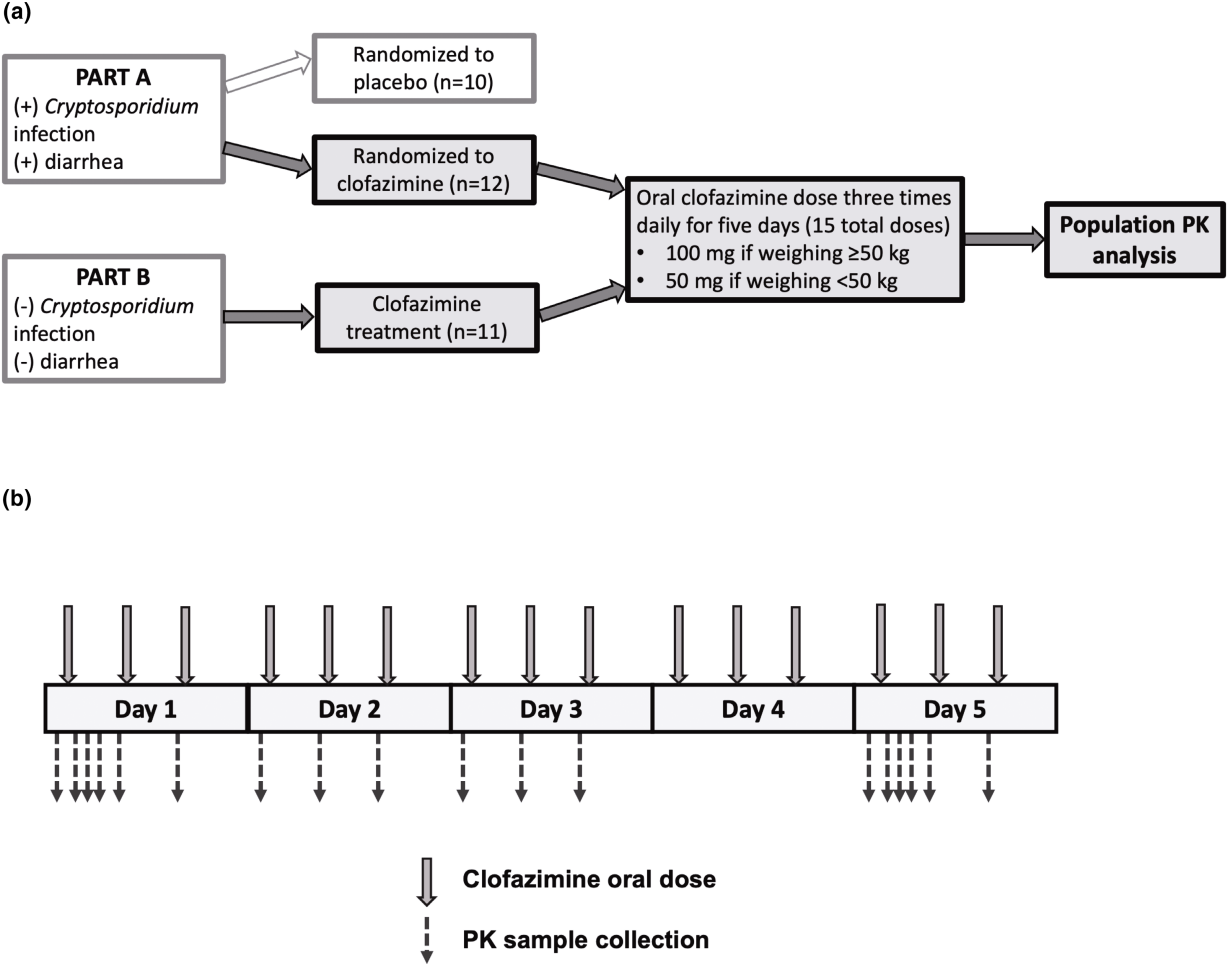
Overview of clofazimine phase IIa clinical trial pharmacokinetic (PK) substudy. (a) The trial consisted of two parts, Parts A and B. Part A participants were HIV‐infected, tested positive for *Cryptosporidium* infection by qPCR, and had persistent diarrhea at enrollment. Part A participants were randomly assigned at approximately 1:1 to receive either clofazimine (*n* = 12) or placebo (*n* = 10) orally three times daily (t.i.d.) for 5 consecutive days. Part B participants were *Cryptosporidium* negative and diarrhea free at enrollment. All Part B participants (*n* = 11) received clofazimine orally t.i.d. for 5 days. The dose of clofazimine was determined by each participant's body weight at enrollment. Participants who weighed greater than or equal to 50 kg received 100 mg clofazimine each dose, whereas those who weighed less than 50 kg received 50 mg clofazimine each dose. Only data from Part A and Part B participants who received clofazimine treatment (*n* = 23) were included for the population PK analysis. (b) Dosing and sampling schemes for Part A and Part B participants who received clofazimine treatment. Oral clofazimine was administered at approximately 5 a.m., 11 a.m., and 6 p.m. on study Days 1–5. Blood samples were collected for PK measurements on Day 1 before the first dose; 2, 3, and 4 h after the first dose, and immediately before the second and third doses. On Days 2 and 3, predose blood samples were collected for every dose. On Day 5, predose samples for every dose and 2, 3, and 4 h post–first dose samples were taken.

Detailed description of the protocol and clinical outcomes of the study have been published previously.^23^, ^24^ This single‐center, randomized, double‐blind, placebo‐controlled phase IIa clinical trial was conducted in Blantyre, Malawi (National Clinical Trial no.: 03341767). Written informed consent was received from all study participants, and the study protocol was approved by relevant regulatory and ethics committees. The study consisted of two parts, Parts A and B (Figure 1a). Eligible participants for Part A were infected with HIV, aged between 18 and 65 years, weighed more than 35.4 kg, received antiretrovirals for at least 1 month, had ≥14 days of diarrhea, and tested positive for *Cryptosporidium* infection by quantitative polymerase chain reaction (qPCR). Part A participants were randomly assigned to receive either clofazimine (*n* = 12) or placebo (*n* = 10). Part B participants (*n* = 11) were HIV infected but did not have *Cryptosporidium* infection or diarrhea. Part B participants were matched to the first 10 Part A participants based on demographic criteria age, sex, and weight to minimize confounding as the result of an imbalanced distribution of potential confounders between the two groups.^24^ All Part B participants received clofazimine.

Clofazimine (a Lamprene® product from Novartis Pharmaceuticals Corporation) was administered orally with food three times daily at approximately 5 a.m., 11 a.m., and 6 p.m. for 5 days (Figure 1b). Every study participant received peanut‐based Plumpy'Nut (Nutriset) supplement 30 min before each dose, which was administered at least 1.5 h before the next anticipated meal.^24^ Participants who weighed ≥50 kg received 100 mg clofazimine, and those <50 kg received 50 mg clofazimine per dose. After the 5‐day drug‐dosing period, patients were followed up by a visit at 19–24 days after the last dose and a final visit at 41–55 days after the last dose.

### Data collection and analysis

Blood samples were collected at screening for CD4+ T cell count and HIV viral load quantification. Additional blood samples were taken for PK measurements on Day 1 before the first dose; 2, 3, and 4 h after the first dose; and immediately before the second and third doses (Figure 1b). On Days 2 and 3, predose blood samples were collected for every dose. On Day 5, predose samples for every dose and 2, 3, and 4 h post–first dose samples were taken. Clofazimine concentrations were measured using a liquid chromatography–tandem mass spectrometry by Q2 Solutions. The assay methods were validated for clofazimine quantification in human plasma within the range of 1.0–1000 ng/mL according to US FDA guidelines.

Stool samples were assessed to determine the presence and severity of diarrhea three times a day before each dose from Days 1 to 5, and once on Day 6 before discharge. Diarrhea, if present at an assessment, would result in a severity grade greater than 0, with Grade 1 representing mild diarrhea and Grade 2 representing severe diarrhea. Daily maximum diarrhea grade was the highest grade observed in three assessments (second and third assessments of the specified study day and the first assessment of the next day), whereas overall maximum diarrhea grade was the highest grade observed among all assessments for each participant. Each assessment with diarrhea counted as one episode of diarrhea. Therefore, up to three episodes of diarrhea could be reported in a day during which stool sample data were collected.

### Modeling software

Exploratory statistical and graphical analyses were conducted in R (Version 4.1.1, R project [http://www.r‐project.org/]). Population PK model development and analysis was performed with Phoenix NLME (version 8.3.5, Pharsight, Certara Inc.) using the first‐order conditional estimation‐extended least squares method.

### Population PK structural model development

Selection of the structural model was based on the following factors: minus two log likelihood (−2LL), Akaike's information criterion, theta correlation matrix, relative standard error (RSE) of parameter estimates, and goodness‐of‐fit (GOF) diagnostic plots. One‐, two‐ and three‐compartment models with first‐order absorption and linear elimination were first compared to identify the simplest structural model that best captured the observed data (Table S1). Absorption lag time (T_lag_) was then introduced to the most parsimonious model to determine whether significant improvement to the model was made based on changes in the objective function value (OFV) as determined by ∆−2LL. Lastly, the inclusion of a relative bioavailability (F_rel_) function, where the typical value of bioavailability (F) was fixed at 1, was assessed.

Additive, proportional, combined proportional and additive, and power residual error models were tested. A combined proportional and additive residual error model was selected as it best fitted the distribution of the residual errors (Equation 1).

(1) $$C_{\mathrm{obs},\mathrm{ij}}=C_{\text{pred,}\text{ij}}+C_{\text{pred},\mathrm{ij}}\times \varepsilon_{1,\mathrm{ij}}+\varepsilon_{2,\mathrm{ij}}$$

$\varepsilon_{i}$ is the unexplained residual variability for the plasma concentration at the *j*th timepoint in subject *i*.

Interindividual variability (IIV) in PK parameters was evaluated using an exponential model with a mean of zero and a variance of $\omega^{2}$, in line with the PK parameters' log‐normal distributions (Equation 2).

(2) $${\mathrm{Par}}_{i}=\theta_{\mathrm{pop}}\times e^{\eta_{i}}$$

In Equation 2, ${\mathrm{Par}}_{i}$ is the estimated PK parameter for the *i*th subject and $\theta_{\mathrm{pop}}$ is the typical value of that parameter in a population. The random effect $\eta_{i}$ describes the amount by which the *i*th subject's parameter value deviates from the population mean.

Interoccasion variability (IOV) was evaluated for F, apparent systemic clearance (CL/F), apparent central compartment volume of distribution (V/F), apparent intercompartmental clearance (Q/F), and apparent peripheral compartment volume of distribution (V2/F). The occasions were defined by the visits during which PK samples were collected. There were eight visits in total. Visits 1 to 6 correspond to Days 1 to 6 of the study. Visit 7 corresponds to the first follow‐up visit at approximately 19–24 days after the last dose. Visit 8 represents the final follow‐up visit at 41–55 days after the last dose.

### Covariate selection

Subject demographic and disease‐related characteristics were selected to be screened based on clinical interest, mechanistic plausibility, and any potential correlation with PK parameters suggested by trends in eta scatter plots (Table 1). The candidate covariates were classified into categorical and continuous covariates. Age and body weight were tested as continuous demographic covariates. Disease (HIV or *Cryptosporidium* infection)‐related continuous covariates assessed included CD4+ T cell count at screening, HIV viral load at screening, daily number of diarrhea episodes, and total number of diarrhea episodes. Disease‐related categorical covariates tested included study part assignment (A or B), number of diarrhea episodes across all assessments (no diarrhea, ≤7 episodes of diarrhea, and >7 episodes of diarrhea), presence of diarrhea on Day 1 (yes or no), daily maximum diarrhea grade (0, 1, or 2), and overall maximum diarrhea grade (0, 1, or 2). The effects of these covariates were tested on the PK parameters absorption constant (k_a_), F, CL/F, V/F, and Q/F. Continuous covariates were normalized by the mean or median of observed values when appropriate and incorporated into the base model by either the linear or the power model based on the level of improvement in model fit (Equations 3 and 4). Categorical covariates were incorporated in a proportional shift model (Equation 5). An exception to this rule is the evaluation of covariates on F. Categorical covariates were included on F using an exponential form (Equation 6).

(3) $${\mathrm{Par}}_{i}=\theta_{0}+\theta_{1}\times {\mathrm{Cov}}_{i}$$

(4) $${\mathrm{Par}}_{i}=\theta_{0}\times {{\mathrm{Cov}}_{i}}^{\theta_{1}}$$

(5) $${\mathrm{Par}}_{i}=\theta_{0}\times \left(1+\theta_{1}|\left({\mathrm{Cov}}_{i}=1\right)+\theta_{2}|\left({\mathrm{Cov}}_{i}=2\right)+\ldots +\theta_{k}|\left({\mathrm{Cov}}_{i}=k\right)\right)$$

(6) $$F_{i}=1\times \exp\left({\eta F}_{i}+\theta_{1}|\left({\mathrm{Cov}}_{i}=1\right)+\theta_{2}|\left({\mathrm{Cov}}_{i}=2\right)+\ldots +\theta_{k}|\left({\mathrm{Cov}}_{i}=k\right)\right)$$

**TABLE 1:** Stepwise covariate search summary.

Model	Covariate added/Removed	‐2(LL)	∆‐2(LL)	#Parms	*p* Value	Compare with
1^a^		−1660.7		13		
2	HIV viral load on Q	−1666.1	−5.4	14	0.020	1
3	HIV viral load on F	−1668.8	−8.1	14	0.004	1
4	HIV viral load on CL	−1661.8	−1.1	14	0.291	1
5	Presence of diarrhea on Day 1 on F	−1695.3	−34.6	14	4.09 × 10^−9^	1
6	Presence of diarrhea on Day 1 on CL	−1667.4	−6.7	14	0.010	1
7	Presence of diarrhea on Day 1 on k_a_	−1662.2	−1.6	14	0.210	1
8	Categorical variable of diarrhea episode^b^ on CL	−1667.6	−7.0	15	0.031	1
9	Categorical variable of diarrhea episode^b^ on F	−1695.6	−34.9	15	2.58 × 10^−8^	1
10	Daily max diarrhea grade on F	−1667.7	−7.1	15	0.029	1
11	Daily max diarrhea grade on CL	−1661.3	−0.6	15	0.746	1
12	Daily max diarrhea grade on k_a_	−1663.2	−2.6	15	0.278	1
13^c^	Overall max diarrhea grade on F	−1700.4	−39.8	15	2.31 × 10^−9^	1
14	Study part on CL	−1662.4	−1.8	14	0.185	1
15	Study part on F	−1670.9	−10.2	14	0.001	1
16	Age on V	−1663.3	−2.6	14	0.104	1
17	CD4 count on F	−1667.4	−6.7	14	0.009	1
18	Daily number of diarrhea episodes on F	−1666.5	−5.8	14	0.016	1
19	Daily number of diarrhea episodes on CL	−1660.5	0.17	14	–	1
20	Daily number of diarrhea episodes on k_a_	−1660.7	−0.1	14	0.794	1
21	Total number of diarrhea episodes on F	−1675.5	−14.8	14	1.16 × 10^−4^	1
22	Weight on V	−1663.4	−2.7	14	0.099	1
23^d^	Overall max diarrhea grade on F	−1767.1		15		
24	Presence of diarrhea on Day 1 on F	−1767.1	0.0	16	0.970	23
25	Presence of diarrhea on Day 1 on k_a_	−1768.1	−1.0	16	0.306	23
26	Daily max diarrhea grade on k_a_	−1769.5	−2.4	17	0.295	23
27	Overall max diarrhea grade on k_a_	−1771.5	−4.4	17	0.111	23
28	Age on V	−1769.2	−2.1	16	0.145	23
29	Age on k_a_	−1767.3	−0.2	16	0.633	23
30	Daily number of diarrhea episodes on k_a_	−1767.3	−0.2	16	0.692	23
31	Categorical variable of diarrhea episode^b^ on F	−1767.1	0.0	17	0.978	23
32	Weight on V	−1769.0	−1.9	16	0.164	23
33	Weight on CL	−1767.1	0.0	16	0.860	23
34	Remove overall max diarrhea grade on F	−1724.73	42.3671	13	6.31 × 10^−10^	23

Abbreviations: #Parms, number of parameters in the model; *∆*‐2(LL), −2(LL) of current model minus −2(LL) of the reference model; −2(LL), −2 × log(likelihood) for each model; CD4, CD4+ T cell; CL, systemic clearance; F, bioavailability; k_a_, absorption rate constant; max, maximum; Q, intercompartmental clearance; V, central compartment volume of distribution.

^a^ Base model.

^b^ The categorical variable of diarrhea episode classifies the total number of diarrhea episodes across all assessments into three categories: no diarrhea, ≤7 episodes of diarrhea, and >7 episodes of diarrhea.

^c^ Selected after first round of forward addition.

^d^ Final model. Model 13 was rerun with all *n* = 23 to include the subject without HIV viral load data.

$\theta_{0}$ in Equations ((3), (4), (5)) represents the typical parameter estimate for the reference group or the baseline. $\theta_{1}$ to $\theta_{k}$ estimate the covariate effect, describing how a parameter changes with the covariate tested. The subscript *i* represents the *i*th subject. *Cov* represents the covariate of interest. For categorical covariates (Equations 5 and 6), all possible categories for the covariate of interest are denoted by numbers 1 to *k*. For example, if participant *i* falls under Category 2 for the categorical covariate of interest, Equation (5) would reduce to ${\mathrm{Par}}_{i}=\theta_{0}\times \left(1+\theta_{2}\right)$ because the condition (${\mathrm{Cov}}_{i}=2)$ holds true.

Candidate covariates were evaluated one at a time using forward addition followed by backward elimination. The threshold for inclusion of a covariate during the forward addition process was set at *p* < 0.01. When multiple covariates satisfied this criterion, the most significant covariate was chosen. Only data from 22 subjects (388 PK samples) were used in the first round of forward addition because HIV viral load information was missing for one participant in Part B. After having determined that HIV viral load was not the most significant covariate to introduce to the structural model and that no appreciable covariate effect by HIV viral load remained after the first selected covariate had been added to the model, subsequent covariate selection steps were performed with the full data set (*n* = 23; 428 PK samples). *p* < 0.001 was set as the threshold for backward elimination. The final inclusion of a covariate was further confirmed by at least a 20% decrease in IIV in the PK parameter of interest.

### Model evaluation

**FIGURE 2:**
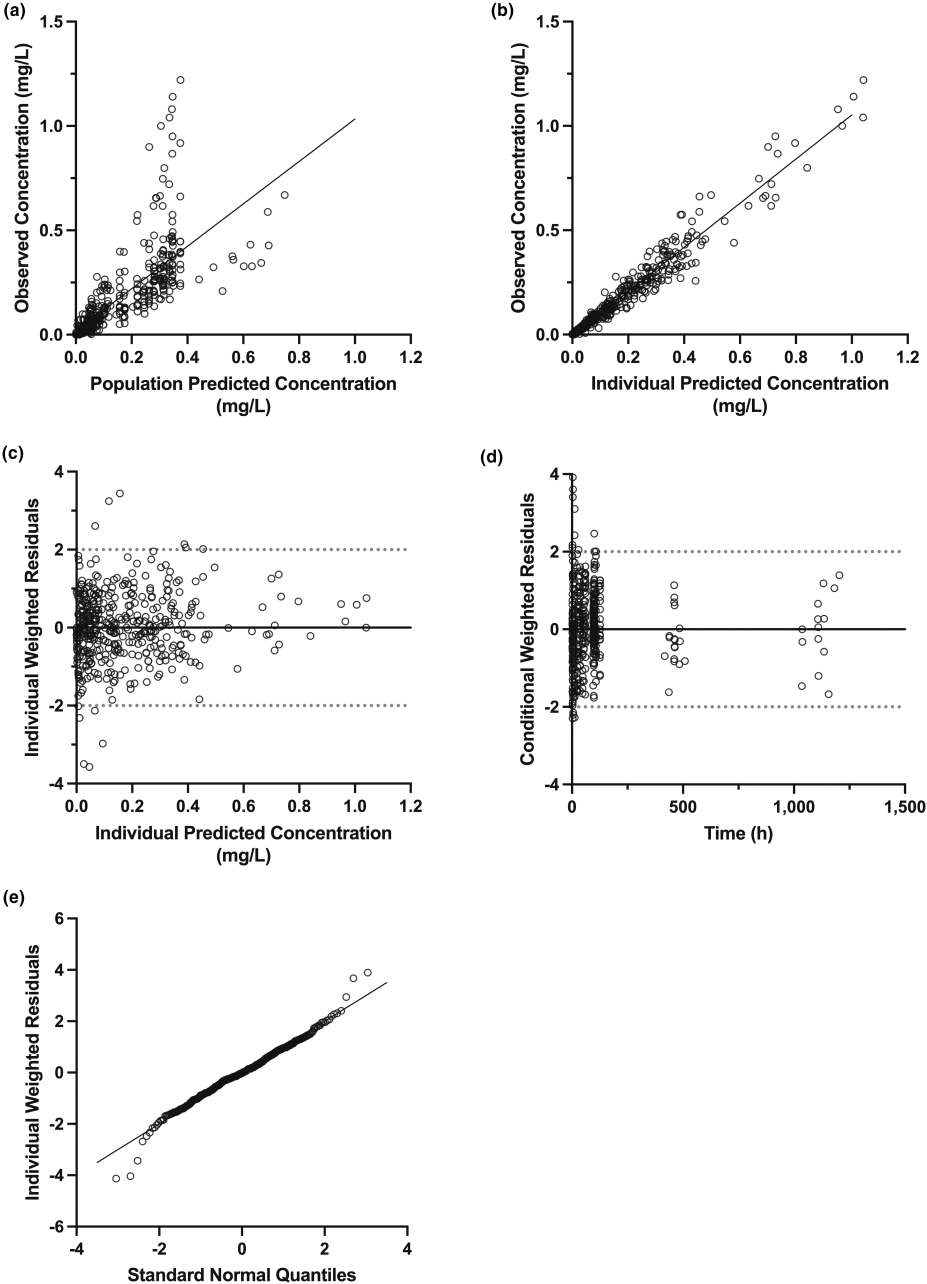
Goodness‐of‐fit plots of the final population pharmacokinetic model for clofazimine. (a) Observed plasma concentration against population predicted concentration, (b) observed plasma concentration against individual predicted concentration, (c) individual weighted residuals against individual predicted concentrations, (d) conditional weighted residuals against time, and (e) quantile–quantile plot of individual weighted residuals.

**FIGURE 3:**
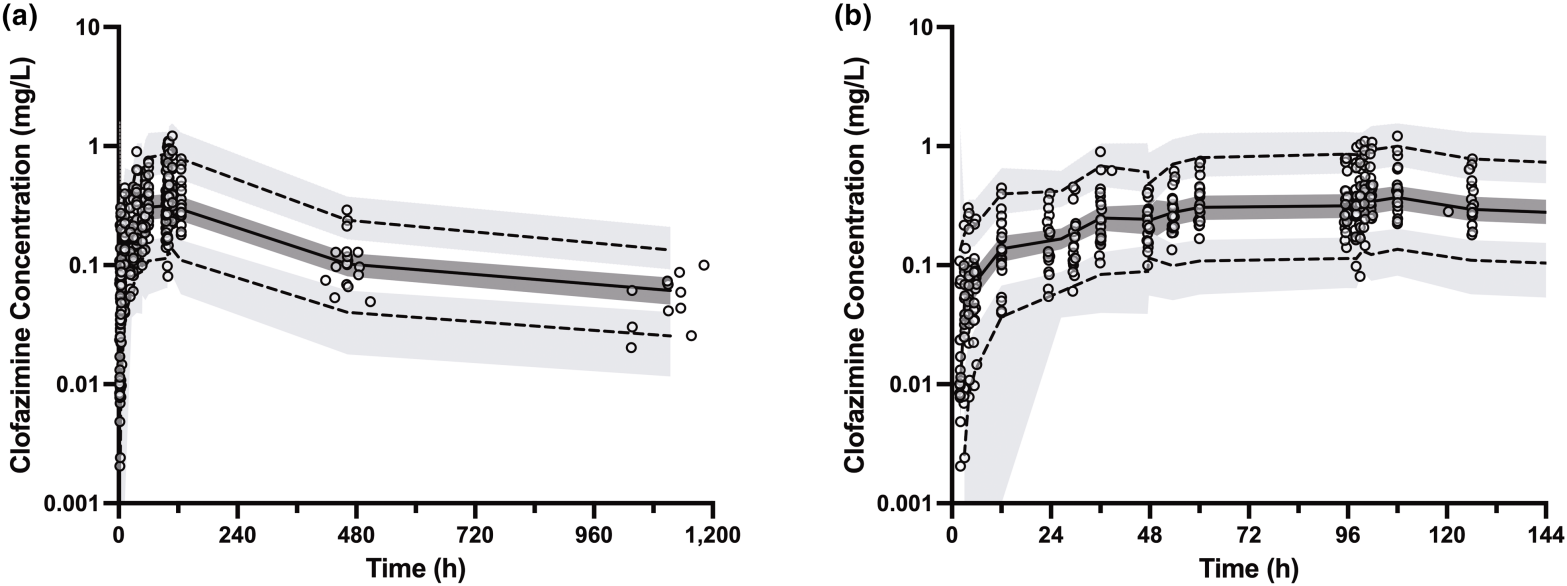
Prediction‐corrected visual predictive check for clofazimine concentration versus time spanning (a) the total duration of the study including the follow‐up visits and (b) the initial 6 days of the study. Open circles represent prediction‐corrected observed plasma concentrations. The observed 5th and 95th percentiles are depicted by dashed lines, and the observed median is represented by the solid line. The shaded regions represent the corresponding model‐predicted 90% confidence intervals around the predicted 5th and 95th percentiles and the median, based on 1000 simulations.

The final population PK model was evaluated with GOF diagnostic plots, bootstrap resampling analysis, prediction‐corrected visual predictive checks (pcVPC), and case deletion diagnostics. The GOF plots examined included observed concentrations (DV) versus population‐predicted concentrations, DV versus individual‐predicted concentrations (IPRED), individual weighted residuals (IWRES) versus IPRED, conditional weighted residuals versus time, and quantile–quantile plot of IWRES (Figure 2). Final model stability was assessed using the nonparametric bootstrap method with 1000 replicates. The final model's ability to reproduce the central tendency and the variability of the observed data was inspected by pcVPC where 1000 replicates were simulated using the fixed‐ and random‐effect estimates from the final model (Figure 3). Furthermore, pcVPC stratified by the covariate overall maximum diarrhea grade was performed to confirm final model performance for these subsets of the population (Figure S1). Lastly, case deletion diagnostics were performed to assess the presence and impact of influential subjects on model parameter estimation (Figure S2).

## RESULTS

### Patient demographics and characteristics

**FIGURE 4:**
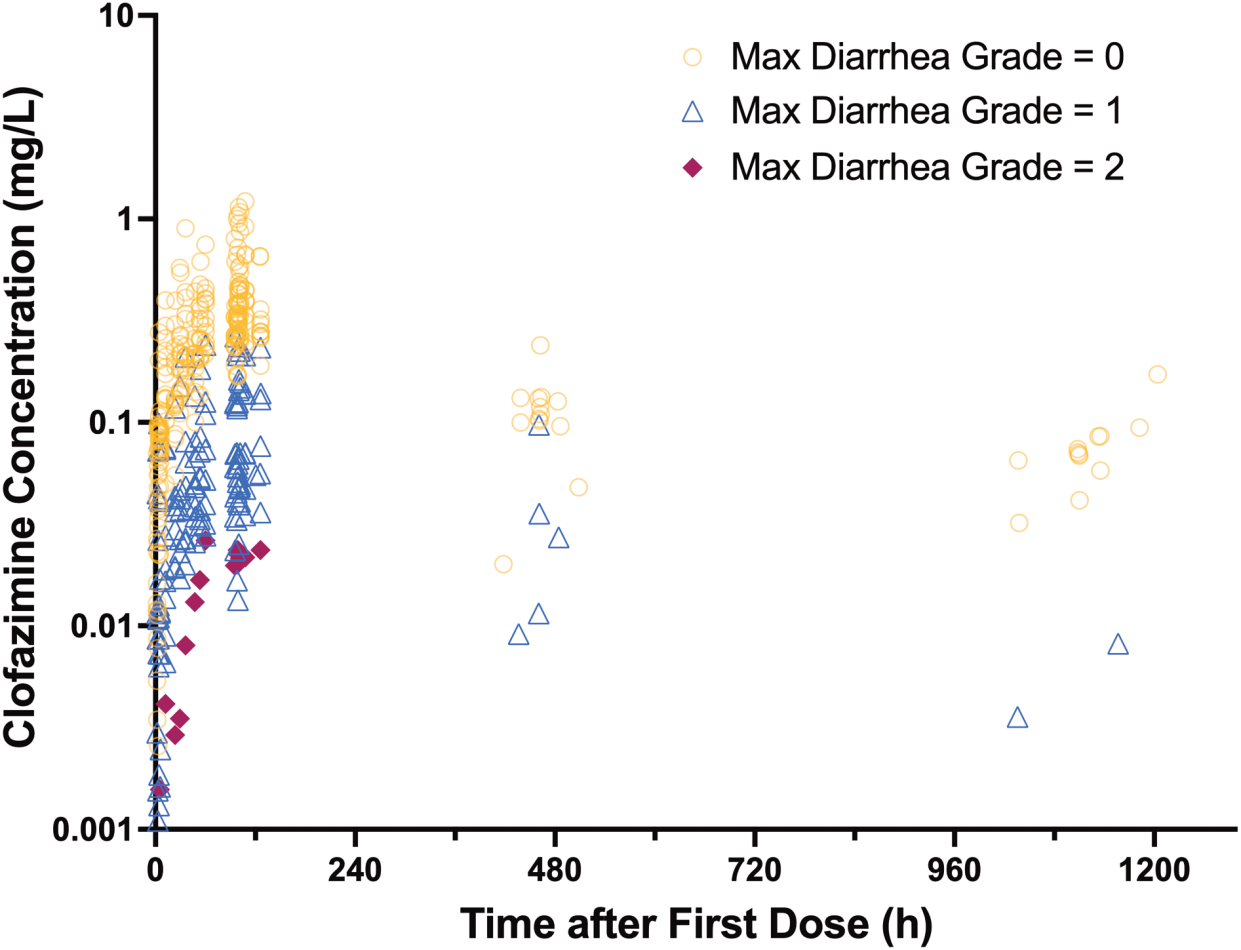
Clofazimine plasma concentrations categorized by overall maximum diarrhea grade. Gold open circles represent pharmacokinetic samples collected from participants who did not have diarrhea for the duration of the study. Dark blue triangles represent samples from participants who only had Grade 1 (mild) diarrhea during the study. Maroon diamonds represent samples from participants who had at least one episode of Grade 2 (severe) diarrhea during the study.

Part B participants were matched to Part A participants based on age, sex, and weight; demographic characteristics were similar between the two groups (Table 2). Although Part A participants were selected based on prolonged diarrhea at screening, only 9 of 12 participants had diarrhea during the 5‐day study period during which clofazimine was administered. No one in Part B had diarrhea during the study. Clofazimine PK data, grouped by the overall maximum diarrhea grade, are plotted in Figure 4. Compared with Part B participants, Part A participants generally displayed more pronounced signs of immunosuppression with a lower median CD4 count and a higher median HIV viral load (Table 2).

**TABLE 2:** Patient characteristics at baseline for Part A and Part B participants who received clofazimine.

	Part A: HIV+/Cryptosporidiosis+		Part B: HIV+/Cryptosporidiosis−	
	(*n* = 12)		(*n* = 11)	
	Median (Range), *n* (%), or *n*	Mean ± SD	Median (Range), *n* (%), or *n*	Mean ± SD
Age, years	40.5 (30, 51)	39.75 ± 7.79	43 (31, 64)	44.09 ± 9.65
Weight, kg	45.45 (36.6, 52)	45.11 ± 4.70	47 (41, 53)	46.96 ± 3.24
Participants				
Male	8 (66.7)		7 (63.6)	
Female	4 (33.3)		4 (36.4)	
HIV viral load, copies/mL^a^	139,819 (26,366, 889,360)	241,981.50 ± 262,806	0 (0, 2683)	275.80 ± 846.13
CD4, cells/μL	23 (3, 93)	25.33 ± 24.44	419 (125, 816)	454.18 ± 227.38
Participant overall max diarrhea grade				
0	3 (25)		11 (100)	
1	8 (66.7)		0 (0)	
2	1 (8.3)		0 (0)	
Participants who received 100 mg/50 mg clofazimine	2/10		1/10	

Abbreviation: CD4, CD4+ T cell; HIV, human immunodeficiency virus.

^a^ HIV viral load data were missing for one Part B participant. Therefore, HIV viral load data at baseline were summarized for *n* = 10 Part B participants.

### Structural PK model

A two‐compartment model with lag time, first‐order absorption, and linear elimination was selected based on the law of parsimony (Table 3). One‐ and three‐compartment models with first‐order absorption and elimination were examined in addition to a two‐compartment model. Whereas the one‐compartment model fell short to describe the biexponentiality in the clofazimine PK profile, the three‐compartment model did not significantly improve the model fit (∆OFV = 0.2) over the two‐compartment model (Table S1).

**TABLE 3:** Base and final population PK model parameter estimates for clofazimine and bootstrap validation (*n* = 1000).

	Base Model			Final Model			Bootstrap	
	Estimate	RSE (%)	Shrinkage	Estimate	RSE (%)	Shrinkage	Mean	95% CI
Parameter								
k_a_ (1/h)	0.601	38.8		0.625	21.0		0.671	0.330 to 1.25
T_lag_ (h)	1.83	3.02		1.83	2.82		1.83	1.72 to 1.91
F	1 (fixed)			1 (fixed)			1 (fixed)	
CL/F (L/h)	7.85	31.9		3.71	10.0		3.72	2.89 to 4.59
V/F (L)	993	30.5		473	10.7		477	350 to 639
Q/F (L/h)	39.5	27.3		18.2	13.2		18.4	13.1 to 24.6
V2/F (L)	7416	23.4		3434	8.07		3424	2685 to 4354
Between‐subject variability								
IIV on V/F (%)	15.2	82.3	0.661	3.46	15.1	0.915	3.48	3.05 to 3.90
IIV on Q/F (%)	45.0	80.8	0.228	45.0	32.4	0.214	42.7	19.1 to 74.4
IIV on k_a_ (%)	161	42.7	0.103	149	28.4	0.105	143	73.8 to 303
IIV on F (%)	137	26.0	0.0308	37.5	35.1	0.151	33.7	9.21 to 49.2
Interoccasion variability on F (%)	35.7	38.4	0.502	36.8	32.8	0.478	37.0	21.9 to 54.3
Covariate effect								
Overall max diarrhea grade = 1				−1.80	11.2		−1.80	−2.25 to ‐1.35
Overall max diarrhea grade = 2				−3.08	3.55		−3.08	−3.38 to ‐2.75
Residual unexplained variability (Additive + Multiplicative model)								
σ	0.00273	47.9	0.129	0.00268	42.0	0.125	0.00269	0.00100 to 0.00532

Abbreviations: CI, confidence interval; CL, systemic clearance; IIV, interindividual variability; k_a_, absorption rate constant; max, maximum; Q, intercompartment clearance; RSE, residual standard error; T_lag_, lag time; V, central compartment volume of distribution; V2, peripheral compartment volume of distribution; σ, an estimate of residual unexplained variability in the plasma concentrations.

Subsequently, T_lag_ and F_rel_ were added to the model as the inclusion of both significantly improved the model fit. IIV was included on k_a_, V/F, Q/F, and F (Table 3). IOV was included on F. The residual error was best captured by a combined proportional and additive residual error model.

### Covariate model

Of all the covariates tested, the covariate overall maximum diarrhea grade was most significantly associated with F and produced the greatest improvement in model performance (Table 1). Therefore, it was chosen as the first covariate to be added to the base structural model.

Although many covariates, when added to the base model in the first round of forward search, produced *p*‐values less than 0.01, they were no longer significant in the second round of forward addition after overall maximum diarrhea grade on F had been added to the model. Not surprisingly, most of these covariates were alternative measures of diarrhea severity, such as Day 1 diarrhea presence and total number of diarrhea episodes (Table 1). The other covariates that were significant in Round 1 but no longer significant in Round 2 were HIV viral load, study part assignment, and CD4 count. Although they were not direct measures of diarrhea severity, all three covariates were correlated with diarrhea status (Figure S3). Therefore, the variability attributed to these covariates in Round 1 of the forward search was possibly accounted for with the addition of overall maximum diarrhea grade as a covariate on F.

For backward elimination, the covariate overall maximum diarrhea grade on F was removed, and this resulted in a significant increase in the OFV (Table 1). Inclusion of overall maximum diarrhea grade on F remarkably reduced the IIV of F from 137% to 37.5%, demonstrating that this covariate was able to account for a substantial portion of the observed variability. Whereas a typical individual without diarrhea during the study has a reference F set to 1, an individual who experienced Grade 1 diarrhea would instead have an F_rel_ of only 0.165 (i.e., sixfold decrease in F), and an individual who experienced Grade 2 diarrhea would have an F_rel_ of 0.0460 (i.e., 22‐fold decrease in F). This extent of F reduction is further supported by comparing maximum clofazimine concentration and area under the clofazimine concentration‐time curve for study participants with different overall maximum diarrhea grades, whereby remarkable differences were observed among the groups (Table S2).

### Population PK model evaluation

The final population PK model estimated all fixed‐effect parameters with high confidence, as shown by the reduction in RSE (Table 3). GOF plots for the final population PK model demonstrate reasonable homoscedasticity and normality for the distribution of residual errors (Figure 2). All weighted residuals, when plotted against time or individual predicted concentrations, suggest that the model is stable. The pcVPC plots support agreement between the simulated and observed data for all groups of participants (Figure 3, Figure S1). The final population model estimates were closely aligned with the mean bootstrap parameter estimates, and all the final model estimates fell within the bootstrap 95% confidence interval (CI) (Table 3). Case deletion diagnostics demonstrated that although a few individuals had more influence on certain parameter estimates than others, the resulting changes in parameter estimates were not pronounced (less than 17%) (Figure S2).

## DISCUSSION

Although oral therapeutics are routinely administered to patients experiencing diarrhea, knowledge about the impact of diarrhea on therapeutic disposition is still limited. To the best of our knowledge, this article is the first to report a significant diarrhea impact on an oral therapeutic's PK and to describe the magnitude of such an impact using a quantitative modeling approach. Our findings not only have important implications for oral clofazimine dosing but also highlight the importance of understanding potential diarrhea impact on oral drug PK in general.

The final model predicted a low clearance (CL/F = 3.71 L/h), large volume of distribution (V/F = 473 L and V2/F of 3434 L), and prolonged half‐life (35.3 days) for clofazimine, in line with current knowledge of clofazimine PK.^28^, ^29^, ^30^ The estimated k_a_ of 0.625 h^−1^ also falls within the k_a_ range of approximately 0.2–1.3 h^−1^ reported by the existing literature.^28^, ^29^ Significant yet highly variable lag time was previously noted for clofazimine.^29^ In line with this notion, the inclusion of T_lag_ into the structural model significantly improved the model (Table S1). Moreover, model evaluation by pcVPC and bootstrap confirmed the stability, precision, and performance of the final model (Table 3, Figure 3, Figure S1). The pcVPC plots show that not only was the trend of the observed data captured by the final model but also the observed variability was accurately depicted (Figure 3). Furthermore, the final model parameter estimates closely matched the mean parameter estimates of the bootstrap analysis and were all within the 95% CI, supporting that the final model estimates were stable and unlikely to be biased due to the inclusion of highly influential points (Table 3).

The application of nonlinear mixed‐effects modeling allowed the estimation of population heterogeneity and the assessment of potential demographic and disease‐associated covariate effects. Previous studies suggested that sex was a significant covariate on peripheral volume of clofazimine distribution, with women having a larger peripheral volume.^28^ However, neither sex nor body weight was a significant covariate in our final model, and this may be attributed to the very narrow range of body weight in our study. Another potential source of variability examined was the dose of clofazimine. The distribution of IWRES for the final model, stratified by clofazimine dose, is comparable between participants who received 50 mg and those who received 100 mg of clofazimine per dose (Figure S4). Although Part B participants were matched to Part A participants based on weight, age, and sex, Part A participants had lower CD4 cell counts and higher HIV viral load compared with their Part B counterparts (Table 2). To examine whether the degree of immunosuppression, HIV‐infection status, or other unidentified underlying differences between Part A and Part B participants impacted clofazimine PK, baseline CD4 count, baseline HIV viral load, and study group assignment were assessed in the covariate selection process. None of these variables remained in the final covariate model as they could not further explain the observed variability (Table 1). The inclusion of the covariate overall maximum diarrhea grade alone accounted for a remarkable level of variability of the observed clofazimine PK. This is evident by comparing the IIV and the RSE of parameter estimates between the base model and the final model in which an approximately 100% reduction in IIV on F and up to fivefold reduction in RSE were achieved for the final model parameters once the overall maximum diarrhea grade covariate had been included (Table 3).

A limitation to this study is the sample size. With a total of 23 participants in the clofazimine PK study, it is possible that there were insufficient data to allow identification of other covariates with higher variability or a less pronounced effect on clofazimine PK. Moreover, only one study participant had an overall maximum diarrhea grade of 2; additional data from more participants experiencing Grade 2 diarrhea are necessary to confidently quantify the magnitude of F reduction associated with an overall maximum diarrhea grade of 2. Furthermore, because of the limited sample size, certain participants can influence PK parameter estimates while the impact is limited (Figure S2). Nevertheless, this study is explorative and the first of its kind for clofazimine.

As a Biopharmaceutics Classification System (BCS) Class II drug, clofazimine is expected to have solubility rate‐limited absorption. Therefore, pathophysiological changes that impact the solubility of the drug may lead to changes in clofazimine bioavailability. Examples of such pathophysiological changes include altered composition of GI fluids, luminal fluid volume, and gut pH.^2^, ^6^, ^31^, ^32^ Accelerated small bowel transit has been reported for HIV‐infected subjects with cryptosporidiosis‐associated diarrhea.^31^ Although a reduction in GI transit time does not directly impact the dissolution process, it leads to less available time for drug dissolution and absorption to take place, potentially resulting in decreased clofazimine bioavailability. Another factor that might have been responsible for the estimated diarrhea impact on clofazimine bioavailability is a change in luminal fluid volume, which can affect both drug dissolution and drug permeation through impacting the drug concentration gradient between the GI lumen and the enterocytes and affecting the thickness and composition of the mucus layer lining the luminal surface. The exact mechanism of how clofazimine bioavailability is impacted, whether a single factor is responsible or if a concerted effort is at play, remains to be elucidated. Mechanistic modeling approaches such as the implementation of physiologically‐based PK (PBPK) modeling may help address this question.^6^ A thorough understanding of cryptosporidiosis disease physiology is also critical for future investigations of the mechanisms behind the diarrhea impact discovered by our current study. Furthermore, mechanistic knowledge is key to making correct dosing decisions across different populations, particularly for drugs such as clofazimine, which may have nonlinear PK at doses higher than what is currently administered clinically, as suggested by a dose‐ranging mouse study.^21^ For these drugs, simply increasing the dose by the same extent by which bioavailability is impacted by disease‐associated covariate(s) may not produce the same targeted drug exposure because bioavailability depends on both physicochemical properties of the drugs as well as physiological parameters of the target population. The solubility limitation for BCS Class II drugs may lead to incomplete dissolution and decreased F at high doses, resulting in a less‐than‐proportional increase in exposure with increasing dose. Therefore, additional modeling approaches that can account for the interaction between drug‐specific parameters and system parameters, such as PBPK modeling, should be used to inform dose predictions.

In conclusion, maximum diarrhea grade over the duration of clofazimine administration was significantly associated with over sixfold reduction in clofazimine oral bioavailability in HIV‐infected adults. This quantitative knowledge is crucial for making correct dosing decisions and may contribute to shaping clinical practices. For instance, alternative treatment options to oral clofazimine may need to be considered for HIV‐infected adults experiencing diarrhea. Our finding also draws attention to studying how diarrhea impacts the PK of other oral therapeutics in other disease areas. Because drug properties and physiological parameters may both be involved in causing a diarrhea impact on oral drug PK, the magnitude of the impact is likely disease specific and drug specific. Therefore, additional studies are needed to identify diseases and drugs that are particularly prone to variability associated with diarrhea. We hope that our findings will inspire future studies of diarrhea impact on oral drug PK and pave the way to informing drug dosing considerations that would expand to benefit additional patient populations.

## AUTHOR CONTRIBUTIONS

C.X.Z., T.M.C., D.H., M.A.G., E.H., P.‐Y.I.T., K.C.J., W.N., D.J.O., J.P., G.V.Q., L.A.S., L.K.B., H.T., N.T., W.C.V.V., and S.L.M.A. wrote the manuscript. C.X.Z., T.M.C., D.H., M.A.G., E.H., P.‐Y.I.T., K.C.J., W.N., D.J.O., J.P., G.V.Q., L.A.S., L.K.B., H.T., N.T., W.C.V.V., and S.L.M.A. designed the research. C.X.Z., T.M.C., D.H., M.A.G., E.H., P.‐Y.I.T., K.C.J., W.N., D.J.O., J.P., G.V.Q., L.A.S., L.K.B., H.T., N.T., W.C.V.V., and S.L.M.A. performed the research. C.X.Z., T.M.C., and S.L.M.A. analyzed the data.

## FUNDING INFORMATION

The work was supported by the Bill & Melinda Gates Foundation (OPP1172544, OPP1160955). The findings and conclusions contained within are those of the authors and do not necessarily reflect positions or policies of the Bill & Melinda Gates Foundation.

## CONFLICT OF INTEREST STATEMENT

The authors declared no competing interests for this work.

## Supporting information


Appendix S1
