# Supplementary material for: Clofazimine pharmacokinetics in HIV‐infected adults with diarrhea: Implications of diarrheal disease on absorption of orally administered therapeutics
Source: CPT Pharmacometrics Syst Pharmacol. 2024 Jan 2;13(3):410–23. doi: 10.1002/psp4.13092 (PMC10941540; doi:10.1002/psp4.13092)
Supplement: Supplementary file 1 — Appendix S1 [file PSP4-13-410-s001.docx]

Supplemental Material: Clofazimine Pharmacokinetics in HIV-Infected Adults with Diarrhea: Implications of Diarrheal Disease on Absorption of Orally Administered Therapeutics

C. X. Zhang^1^, T. M. Conrad^2*^, D. Hermann^3^, M. A. Gordon^4,5^, E. Houpt^6^, P.Y. Iroh Tam^4,7^, K. C. Jere^4,5^, W. Nedi^4^, D. J. Operario^6*^, J. Phulusa^4^, G. V. Quinnan Jr^2^, L. A. Sawyer^2^, L. K. Barrett^1^, H. Thole^4^, N. Toto^7^, W. C. Van Voorhis^1^, and S. L. M. Arnold^1#^

^1^University of Washington, Seattle, Washington, USA

^2^Emmes, Rockville, Maryland, USA

^3^Bill & Melinda Gates Foundation, Seattle, Washington, USA

^4^Malawi-Liverpool Wellcome Research Programme, Blantyre, Malawi

^5^University of Liverpool, Liverpool, UK

^6^University of Virginia, Charlottesville, Virginia, USA

^7^Liverpool School of Tropical Medicine, Liverpool, UK

***Affiliation at the time of this study

**
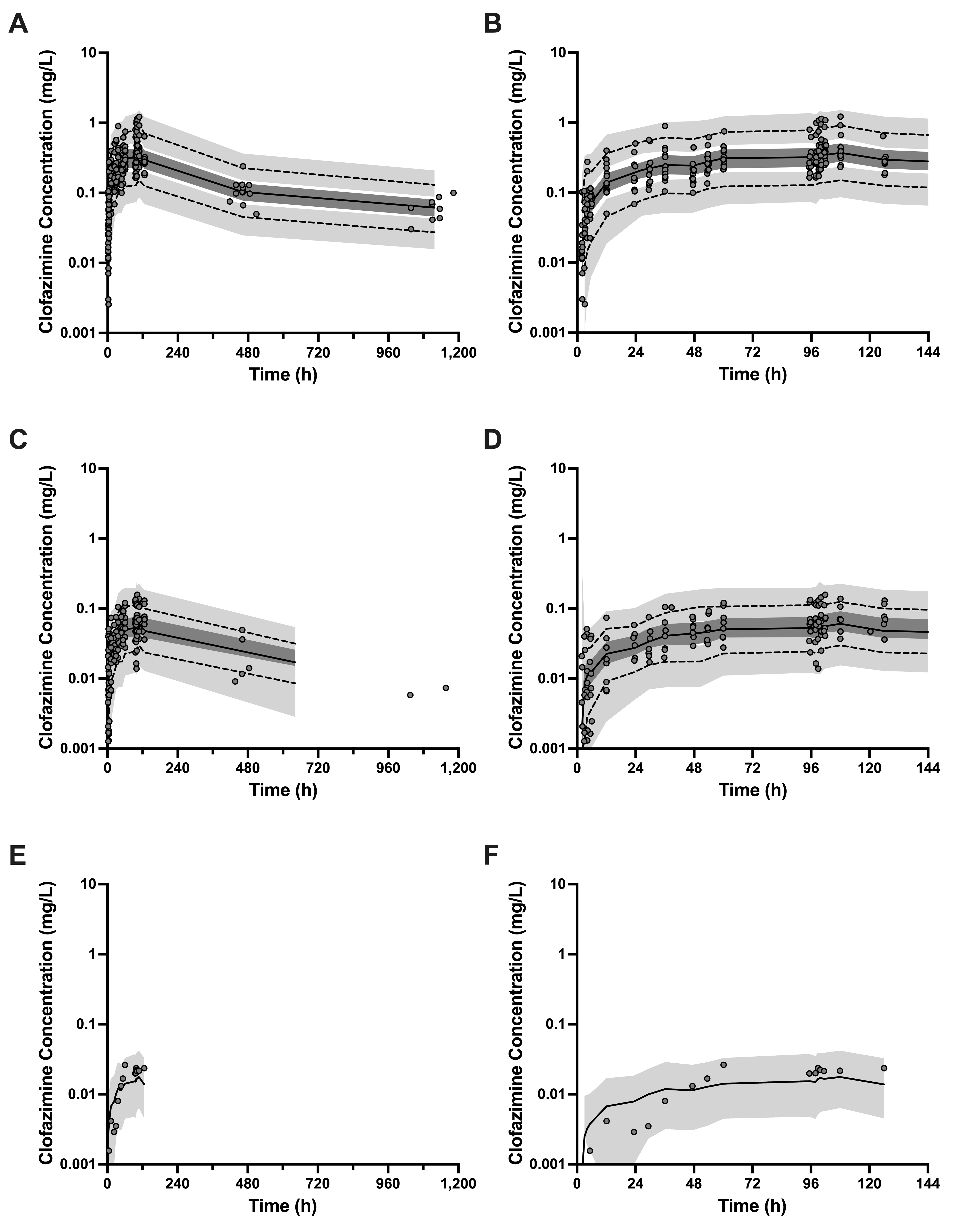
**

**Figure S1** Prediction-corrected visual predictive check for clofazimine concentration versus time stratified by the overall maximum diarrhea grade observed during the study. Diarrhea was absent in participants shown in **A** and **B,** whereas a maximum of grade 1 diarrhea was observed in the group shown in **C** and **D** and a maximum of grade 2 diarrhea was observed in the participant shown in **E** and **F.**  Panels **A, C,** and **E** span the total duration of the study including the follow-up visits, whereas **B, D,** and **F** provide a directed view at the initial 6 days of the study. Grey open circles represent prediction-corrected observed plasma concentrations. The observed 5th and 95th percentiles are depicted by dashed lines, and the observed median is represented by the solid line. The shaded regions represent the corresponding model predicted 90% confidence intervals around the predicted 5th and 95th percentiles and the median, based on 1,000 simulations.

**
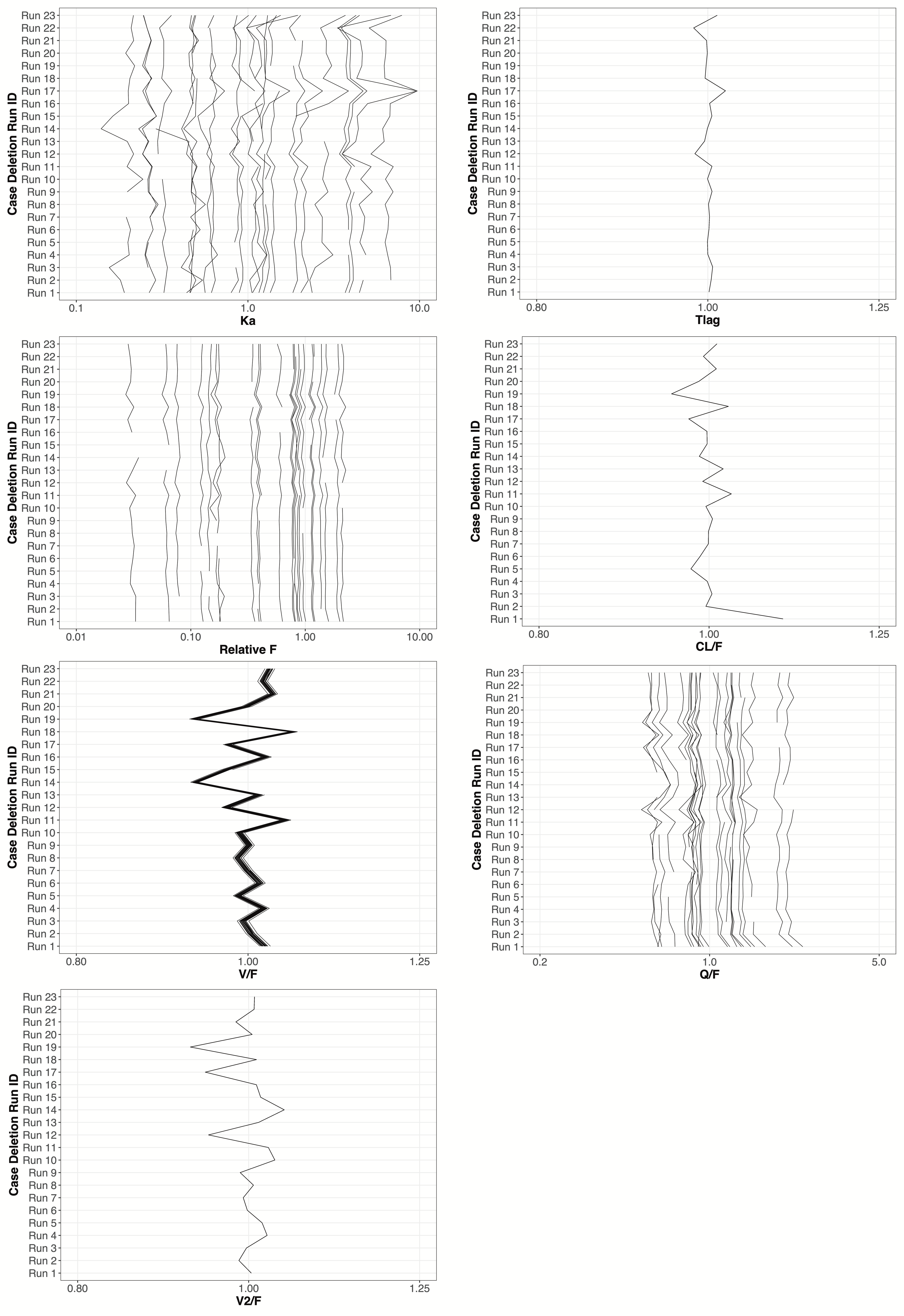
**

**Figure S2** Parallel coordinate plot for diagnosing case deletion runs for pharmacokinetic parameter estimates. Each line in the figures connects values for each subject or the typical value, if IIV was not estimated for that pharmacokinetic parameter, across runs. Pharmacokinetic parameter estimates were normalized by the final model estimate.


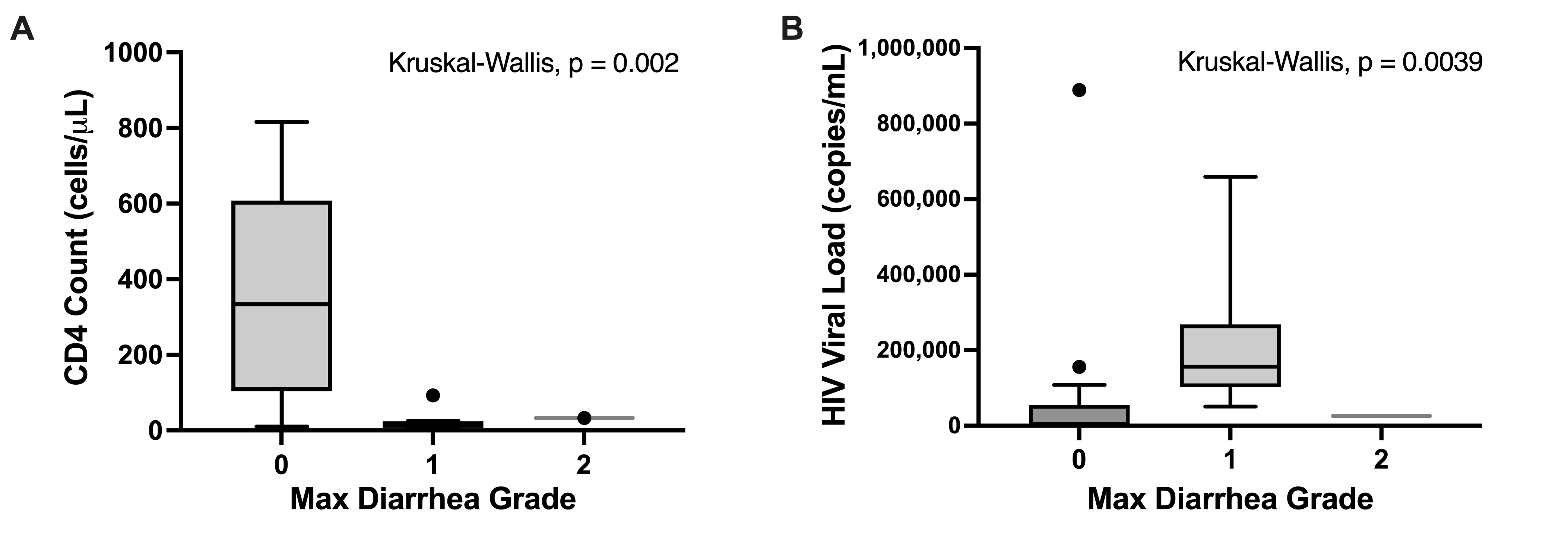


**Figure S3** Box plots of immunosuppression and HIV infection severity associations with overall maximum diarrhea grade over the duration of the study. CD4 count (**A**) and HIV viral load (**B**) were compared among groups of participants without diarrhea (n=14), with maximum grade 1 diarrhea (n=8), and with maximum grade 2 diarrhea (n=1), with p-values computed using the Kruskal-Wallis test.


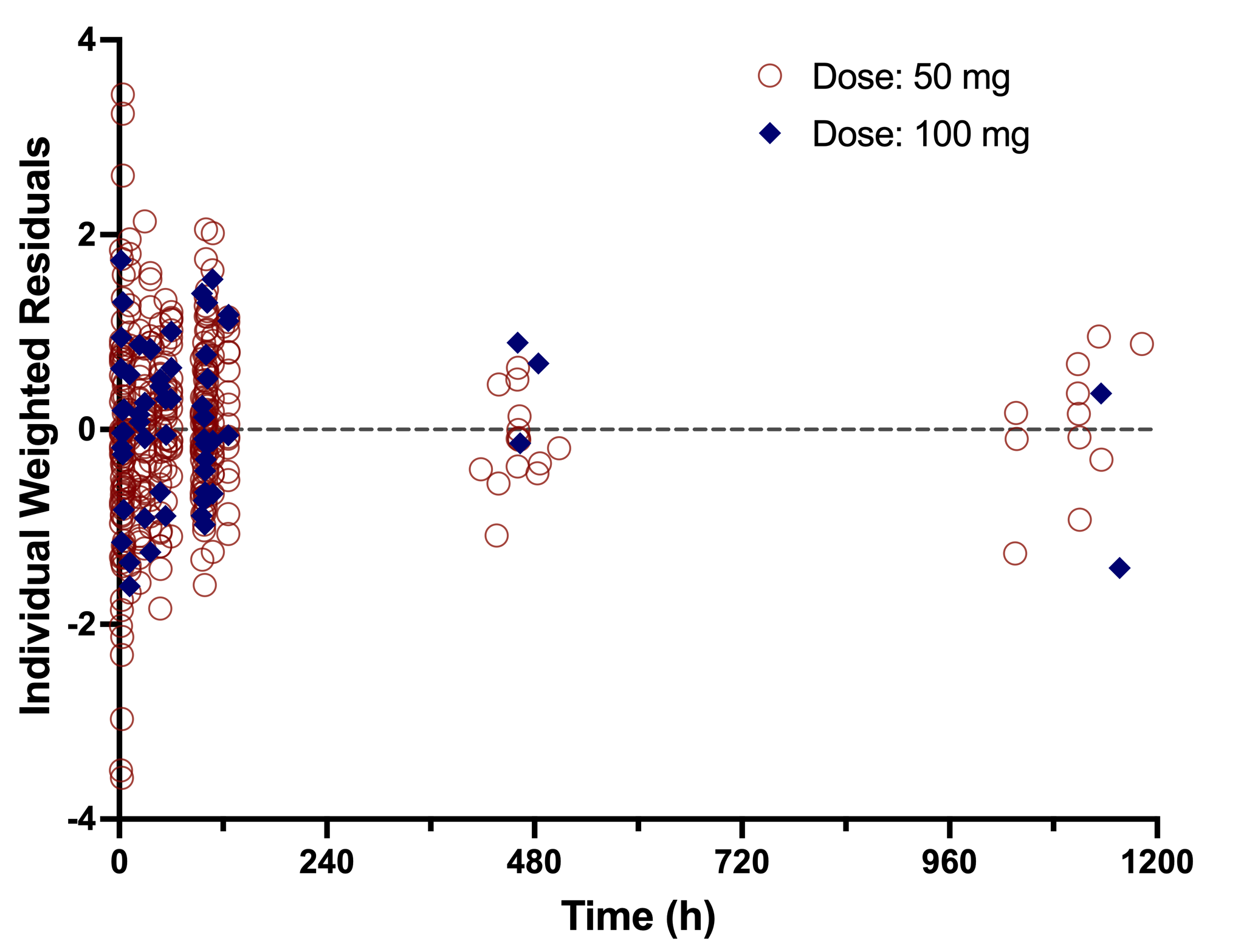


**Figure S4** Individual weighted residuals (IWRES) of the final population pharmacokinetic model, categorized by the dose of clofazimine each participant received, were plotted against time. Open circles represent IWRES comparing observed clofazimine plasma concentrations to final-model-predicted concentrations for participants who received 50 mg of clofazimine per dose. Solid diamonds represent IWRES comparing observed plasma clofazimine concentrations to final-model-predicted concentrations for participants who received 100 mg of clofazimine per dose.

**Table S1** Base model building summary.

| Model | Model Structure | -2(LL) | $\boldsymbol{\Delta}$-2(LL) | #Parms | df | P-value | Compare with |
| --- | --- | --- | --- | --- | --- | --- | --- |
| 1 | 1-compartment | -1165.4 |  | 8 |  |  |  |
| 2 | 2-compartment | -1522.8 | -357.4 | 12 | 4 | 4.39$\times{10}^{-76}$ | 1 |
| 3 | 3-compartment | -1522.5 | 0.2 | 16 | 4 | - | 2 |
| 4 | 2-compartment with T_lag_ | -1628.8 | -106.1 | 14 | 2 | 9.27$\times{10}^{-24}$ | 2 |
| 5* | 2-compartment with T_lag_, F_rel_ | -1693.4 | -64.5 | 15 | 1 | 9.$56\times{10}^{-16}$ | 4 |

Abbreviation: #Parms, number of parameters in the model; -2(LL), $-2\times log(\mathrm{Likelihood})$ for each model; $\boldsymbol{\Delta}$-2(LL), -2(LL) of current model minus -2(LL) of the reference model; df, degrees of freedom; T_lag_, lag time; F, bioavailability

* Selected base model structure

**Table S2** Pharmacokinetic parameters comparison between participants with different overall maximum diarrhea grades. C_max_ and AUC values were normalized by dose.

|  |  | **Overall Max Diarrhea Grade = 0 (n=14)** | | | **Overall Max Diarrhea Grade = 1 (n=8)** | | | **Overall Max Diarrhea Grade = 2 (n=1)** | | |
| --- | --- | --- | --- | --- | --- | --- | --- | --- | --- | --- |
|  |  | **Mean** | **SD** | **%CV** | **Mean** | **SD** | **%CV** | **Mean** | **SD** | **%CV** |
| **Day 1** | **C_max_ (ng/mL/mg)** | 3.99 | 2.08 | 52.0 | 0.755 | 0.408 | 54.0 | 0.0828 | - | - |
|  | **T_max_ (h)** | 15.0 | 7.13 | 47.5 | 22.1 | 4.06 | 18.4 | 12.1 | - | - |
|  | **AUC_0-24_ (ng*h/mL/mg)** | 60.1 | 28.1 | 46.9 | 10.8 | 7.71 | 71.4 | 1.24 | - | - |
| **Day 5** | **C_max_ (ng/mL/mg)** | 10.8 | 5.38 | 49.8 | 1.58 | 0.827 | 52.2 | 0.472 | - | - |
|  | **T_max_ (h)** | 103 | 3.71 | 3.61 | 102 | 3.85 | 3.78 | 98.9 | - | - |
|  | **AUC_96-120_ (ng*h/mL/mg)** | 195.7 | 118.4 | 60.5 | 32.6 | 19.5 | 59.9 | 10.5 | - | - |

Abbreviation: SD, standard deviation; %CV, percent coefficient of variation; C_max_, maximum concentration; T_max_, time at which C_max_ is achieved; AUC, area under the concentration-time curve
